# Sampling of fluid through skin with magnetohydrodynamics for noninvasive glucose monitoring

**DOI:** 10.1038/s41598-021-86931-7

**Published:** 2021-04-07

**Authors:** Tuuli A. Hakala, Alejandro García Pérez, Melissa Wardale, Ida A. Ruuth, Risto T. Vänskä, Teemu A. Nurminen, Emily Kemp, Zhanna A. Boeva, Juha-Matti Alakoskela, Kim Pettersson-Fernholm, Edward Hæggström, Johan Bobacka

**Affiliations:** 1 GlucoModicum Ltd, A.I. Virtasen Aukio 1, 00560 Helsinki, Finland; 2 Laboratory of Molecular Science and Engineering, Faculty of Science and Engineering, Åbo Akademi University, Biskopsgatan 8, 20500 Turku/Åbo, Finland; 3 Skin and Allergy Hospital, Meilahdentie 2, 00250 Helsinki, Finland; 4 Nefrologian Poliklinikka, Helsinki University Hospital, Haartmaninkatu 4, 00029 Helsinki, Finland; 5 Department of Physics, University of Helsinki, Gustaf Hällströmin katu 2, 00560 Helsinki, Finland

**Keywords:** Techniques and instrumentation, Bioanalytical chemistry

## Abstract

Out of 463 million people currently with diabetes, 232 million remain undiagnosed. Diabetes is a threat to human health, which could be mitigated via continuous self-monitoring of glucose. In addition to blood, interstitial fluid is considered to be a representative sample for glucose monitoring, which makes it highly attractive for wearable on-body sensing. However, new technologies are needed for efficient and noninvasive sampling of interstitial fluid through the skin. In this report, we introduce the use of Lorentz force and magnetohydrodynamics to noninvasively extract dermal interstitial fluid. Using porcine skin as an ex-vivo model, we demonstrate that the extraction rate of magnetohydrodynamics is superior to that of reverse iontophoresis. This work seeks to provide a safe, effective, and noninvasive sampling method to unlock the potential of wearable sensors in needle-free continuous glucose monitoring devices that can benefit people living with diabetes.

## Introduction

Diabetes causes 4 million deaths and costs 800 billion USD per year, affecting 463 million people globally. Additionally, 374 million people have impaired glucose tolerance (IGT), a high-risk state for diabetes^1^. By 2030, the number of people with diabetes and IGT is projected to increase to 578 million and 454 million, respectively^2^. Despite the high human, social, and economic cost that diabetes imposes on nations, diabetes awareness remains low and nearly 50% of all people with diabetes remain undiagnosed. Wearable devices for continuous self-monitoring of glucose can play a crucial role in the fight against diabetes by promoting early detection, adequate management, and increased awareness of diabetes. However, widespread adoption of continuous glucose self-monitoring requires further technological developments that improve user-friendliness and performance^3^.

Blood glucose monitoring is essential for the diagnosis and management of diabetes^3–8^. The main drawback of existing technology is the non-continuous mode and discomfort related to invasive blood sampling. Therefore, extreme glucose levels may remain unnoticed, which can compromise the health of diabetic persons. Therefore, much research is currently directed towards the development of noninvasive and wearable glucose sensors and monitoring devices^9–11^.

Lately, there has been significant progress in the development of wearable sensors for continuous monitoring of glucose and other biomarkers in biofluids such as interstitial fluid, sweat, tears, and saliva^12–18^. Among these biofluids, interstitial fluid (ISF) has a similar glucose concentration to blood plasma, while the concentration of glucose in saliva and sweat is much lower^14,54^. From an analytical point of view, ISF is therefore an attractive sample for noninvasive glucose monitoring. GlucoWatch^®^ (Cygnus, Inc.)^19^, which was commercialized as a wearable glucose monitoring device, used reverse iontophoresis to extract ISF through skin. However, the device was withdrawn from the market in the late 2000s, indicating the great scientific and technological challenges related to noninvasive glucose monitoring^12–18^. Despite intensive research and development, the market is still void of a noninvasive glucose monitor^12^.

Measuring glucose concentrations for clinical purposes via noninvasive glucose monitoring has proven to be challenging. In recent years, extraction of ISF by reverse iontophoresis has received relatively little attention for glucose monitoring^20,21^ compared to glucose measurements in sweat^22–28^. Sweat is easily accessible but the sweat rate and chemical composition of sweat varies greatly as a function of physical activity of the person^28^. During physical exercise, sweat production is sufficiently high to allow for microfluidic sampling and monitoring^23–26^. At rest, a sufficiently high rate of perspiration can be attained by locally activating sweat production through iontophoretic delivery of substances such as pilocarpine, acetylcholine, or methacholine into the skin^28^. Other noninvasive approaches include glucose measurements in tears^29,30^, while microneedles represent a minimally invasive option for measurements in ISF^31,32,33,34^. All commercially available devices for continuous glucose monitoring, i.e. Guardian Connect CGM (Medtronic Inc.), Dexcom G6 (Dexcom Inc.), FreeStyle Libre (Abbott Diabetes Care Inc.), and Eversense CGM System (Senseonics Inc.) rely on invasive sampling of ISF using microneedles or subcutaneous implants^25,26,32,35^. This indicates that, in addition to blood, ISF is a relevant biological fluid for glucose measurements.

Here, we present a novel method based on magnetohydrodynamics (MHD) for extraction of interstitial fluid. Magnetohydrodynamics is a physical phenomenon where fluid flow is induced by the Lorentz force generated by external magnetic and electric fields. The same physical mechanism has been used in other biomedical applications, e.g. in micropumps^36–38^ and jet injectors^30,39^. However, to our knowledge MHD is yet to be investigated as a physical mechanism to extract interstitial fluid from the skin.

Using porcine skin as an ex-vivo model, we show that MHD allows faster extraction compared to reverse iontophoresis. Furthermore, we show that the extracted glucose exhibits a linear relationship to the glucose concentration in a hydrogel in contact with the porcine skin, indicating the feasibility to use MHD as a quantitative tool. MHD increases the total amount of extracted glucose by a factor of two and the active extraction by a factor of 13 when compared to reverse iontophoresis. Hence, MHD reduces the amount of energy applied to the skin required for dermal interstitial fluid sampling and therefore potentially reduces the risk of skin reactions at the extraction site. In this instance, this extraction method is not specific to glucose. It could be applied to extract or deliver other diagnostically or therapeutically valuable molecules through the skin.

## Results

### Principle and experimental setup for MHD-based extraction of interstitial fluid

**Figure 1:**
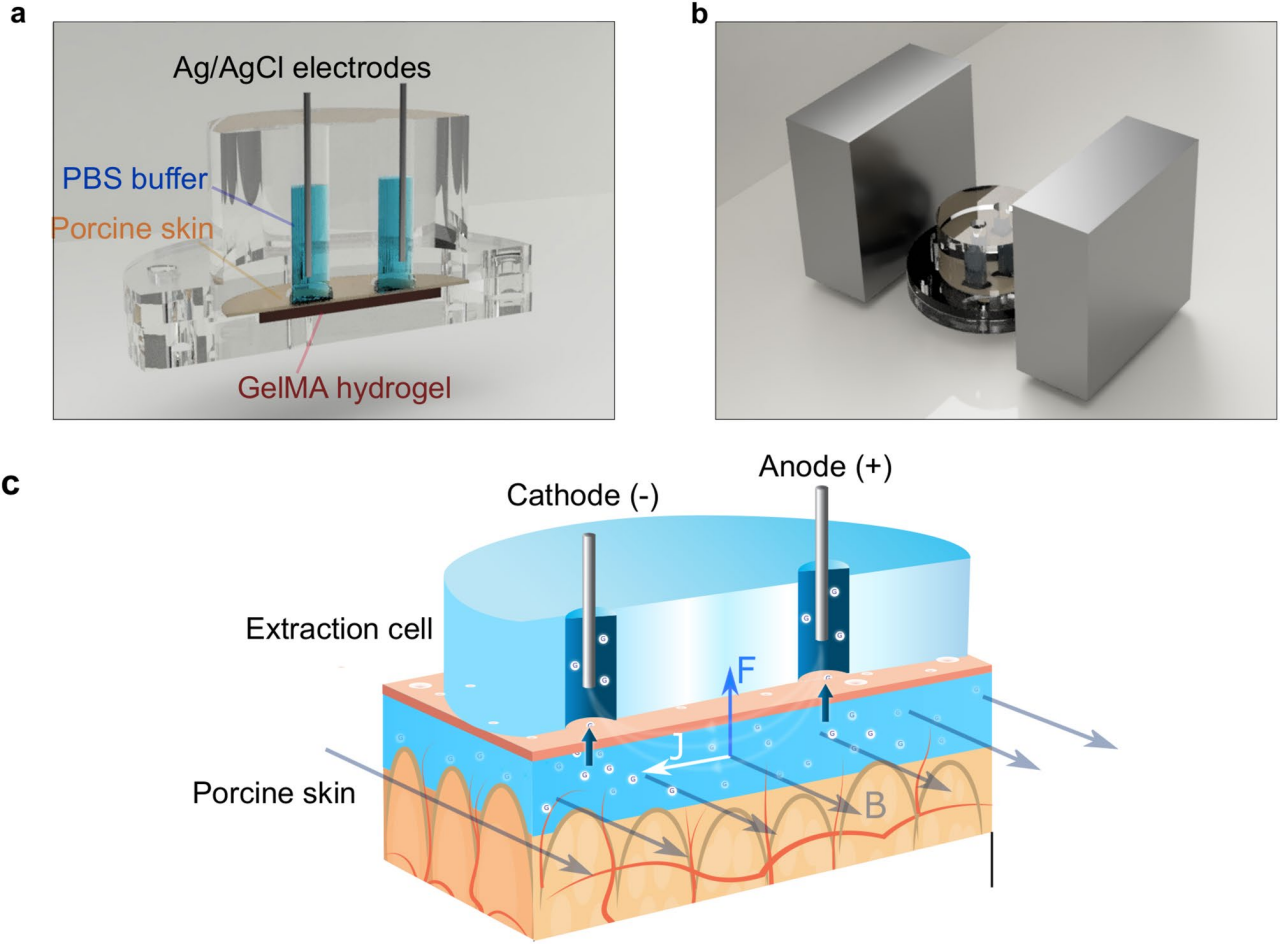
(**a**) 3D model construction of MHD with the extraction cell cut out to reveal the main parts of the cell: GelMA hydrogel at the bottom, porcine skin on top of the hydrogel, electrode wells filled with buffer (PBS, 10 mM, pH 7.4) and Ag/AgCl electrodes (ø = 1 mm, length = 2.5 cm). (**b**) The extraction chamber is positioned between two neodymium magnets (size: 70 mm × 70 mm × 30 mm). (**c**) Schematic picture of glucose extraction using MHD. An electric current is established between the two electrode chambers filled with buffer solution. The electric current induces electro-osmotic flow from the anode through the skin towards the cathode. A current density profile (J) in the presence of a magnetic field (B) generates a Lorentz force (F) that drives the interstitial fluid towards the skin surface.

To demonstrate the efficacy of MHD, we performed experiments using an extraction cell (Fig. 1a) inspired by the work of Ching et al.^40^. We chose to use porcine skin, which is widely employed as a model of human skin for in vitro studies^41,42^. Especially, *stratum corneum* from porcine ear has shown significant correlation to its human counterpart in biophysical properties^42^. To model the deeper skin layers, we used gelatin methacryloyl (GelMA) hydrogel because it mimics the collagen-rich extracellular matrix^43^. We used an extraction cell featuring a lower and an upper chamber. We used an extraction cell featuring a lower and an upper chamber. The lower chamber (Fig. S2a) houses a two-layer skin model constructed from GelMA hydrogel, saturated with a solution of glucose of known concentration (Fig. S2b), and porcine ear skin (Fig. S2c). The upper part of the diffusion chamber, depicted in Figs. 1a and S2s, has three functions. Firstly, it creates a tight seal on the skin model, preventing any leakages. Secondly, it protects the hydrogel and skin from drying. And thirdly, provides a robust experimental setup for glucose extraction depicted in Figs. 1a and S2d. The upper part of the diffusion chamber features two cylindrical electrode wells (⌀ = 6 mm; height = 24 mm; separation = 4 mm) with square openings (l = 7 mm) facing the porcine skin. One electrode well was used as cathodic electrode (−) and one as anodic (+). This was determined by the direction of the applied current. Each well was filled with 400 µl of phosphate buffered saline (PBS, 10 mM, pH 7.4) (Fig. S2e) to conduct the electric current from the anodic Ag/AgCl wire through the skin and to the cathodic wire.

This extraction cell was positioned between two magnets to create a homogenous magnetic field across the cell (Fig. 1b). This magnetic field was combined with electric field to apply MHD extraction through our skin model. The extraction process and forces acting on the interstitial fluid in porcine skin graft are schematically presented in Fig. 1c. The electric field induces electro-osmotic flow from the anode, through the skin, and towards the cathode. The magnetic field orthogonal to the electric field induces a Lorentz force on the interstitial fluid and hence magnetohydrodynamic fluid flow from the deeper skin layers towards the outer skin surface. The extracted fluid accumulates in the electrode wells, and the glucose concentration was determined after each experiment. The experiments were carried out at room temperature and no self heating was detected.

### Comparison of glucose extraction methods

**Figure 2:**
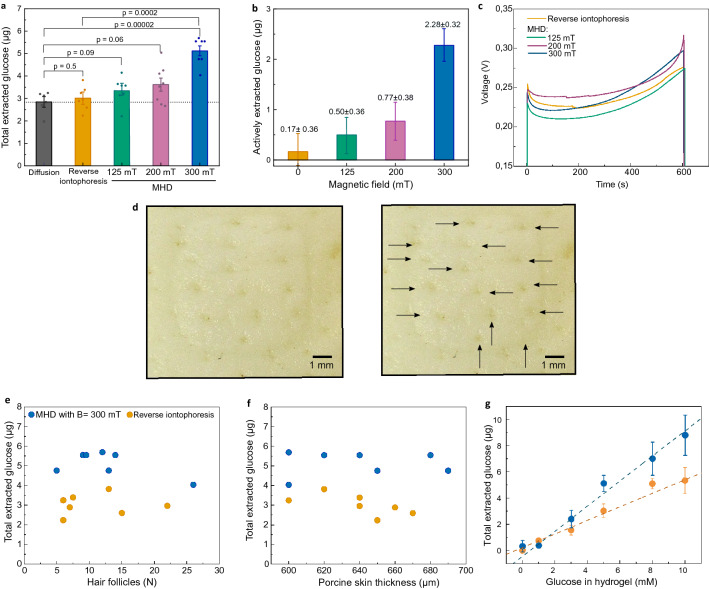
(**a**) Measured amount of glucose collected in the cathodic electrode well after 10 min extraction with passive diffusion, reverse iontophoresis, and MHD using 125 mT, 200 mT and 300 mT magnetic fields. Each extraction was done at room temperature (22 ± 1 °C) using GelMA hydrogel with 5 mM glucose, and an extraction current of 300 µA. P-values were calculated using an unpaired t-test. (**b**) The amount of actively extracted glucose was calculated by subtracting the contribution of passive glucose diffusion. (**c**) Measured voltage curves during extraction. Voltage between the extraction electrodes was measured during each extraction. (**d**–**f**)Amount of extracted glucose in relation to the number of hair follicles and thickness of the skin samples. (**d**) Pictures from a porcine skin sample after extraction at the electrode well area, where the individual hair follicles are marked with arrows. (**e**) Number of hair follicles and total amount of glucose measured (extraction + diffusion) for both iontophoresis and MHD with 300 mT magnetic field. (**f**) Thickness of porcine skin and total amount of glucose measured (extraction + diffusion) for both iontophoresis and MHD with 300 mT magnetic field. (**g**) Total extracted glucose with different glucose concentrations in the GelMA hydrogel (p = 0.0002 from an F-test) (B = 300 mT, I = 300 µA). In figs (**a**–**c**) the bars show the average from at least 6 individual experiments and the error bars represent standard deviation of the mean.

We compared the amount of glucose actively extracted by MHD and reverse iontophoresis against passive diffusion (Fig. 2). The amount of glucose diffusing passively through the skin was measured using the same set-up that was used in the MHD and reverse iontophoresis experiments but without applying electric current or magnetic field. We chose reverse iontophoresis as a reference for active extraction because reverse iontophoresis is the most studied noninvasive method for extraction of glucose and other analytes from the skin^40,44–48^. The amount of glucose extracted and collected in the cathodic electrode well was quantified using a colorimetric assay. We found that a large part of the measured glucose diffused passively to the electrode wells, and no clear difference between reverse iontophoresis and passive diffusion was observed (Fig. 2a). However, there was a substantial increase in the amount of extracted glucose when MHD was applied. In order to determine the contribution of the active transport of glucose by MHD and reverse iontophoresis, the effect of diffusion was subtracted from the extraction data for both reverse iontophoresis and MHD. Accordingly, the estimated amount of glucose extracted actively with MHD (2.28 ± 0.32 µg) was 13 times higher than the amount of glucose extracted actively with reverse iontophoresis (0.17 ± 0.36 µg), when using 10 min extraction time, 300 µA extraction current, and 300 mT for MHD.

The distribution of the current density in the MHD and reverse iontophoresis experiments depends on the size and morphology of the electrode areas, the electrode separation, and the impedance profile of the skin. In the case of MHD, the distribution of the electric current further depends on the strength and distribution of the magnetic field. At constant electric energy deposition and constant electrode contact area, the distribution of the current density in the skin does not significantly affect the extraction efficiency according to the literature of reverse iontophoresis^45,49^. For MHD, the distribution of the current density in the skin determines the distribution of the Lorentz force according to **F** = **J** × **B**. This may allow maximizing the energy efficiency of the extraction by optimizing the electrode morphology and the distribution of the magnetic field. Since the magnetic field was measured inside the empty cathodic electrode well, the exact distribution of the magnetic field across the GelMA hydrogel and the skin was unknown. However, the extraction cell was positioned at the center and in between the magnets, where the magnetic field is substantially homogenous. Furthermore, skin, water, air, and the materials of the set up (i.e. PMMA/Acrylic) feature similar relative magnetic permeabilities (*μ*_r_ ≈ 1). Hence, little distortion of the magnetic field induced by the different materials was expected.

During each extraction, we monitored the voltage between the extraction electrodes to investigate potential discrepancies in electric impedance in the skin samples. Figure 2c shows the measured voltage responses for reverse iontophoresis and MHD with different magnetic fields. For each condition, the voltage stayed relatively constant and the average voltage for each extraction was 0.24 V (Fig. S3). After each experiment, we counted the number of hair follicles inside the area of the electrode well (Fig. 2d) to investigate potential correlation between the number of hair follicles and the amount of extracted glucose. The number of hair follicles was between 5 and 26. However, most skin samples featured 5 to 15 hair follicles. Even though the spread in the number of hair follicles per well area was relatively large, we observed no obvious correlation between the number of hair follicles and extracted glucose (Fig. 2e). Furthermore, we studied whether small differences in skin thickness affect the extraction (Fig. 2f). We chose skin thicknesses between 600 and 700 µm and observed no trends in the amount of glucose extracted with increasing skin thickness. However, the amount of glucose extracted during 10 min depended on the glucose concentration in the GelMA hydrogel (Fig. 2g). This finding indicates that MHD could potentially be used in a quantitative manner with a suitable sensor to measure glucose levels in human interstitial fluid.

### Extraction time optimization

**Figure 3:**
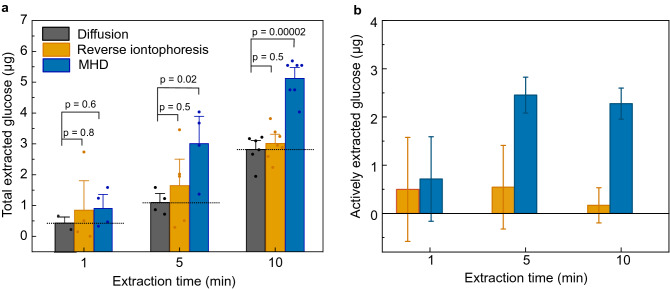
(**a**) The effect of extraction time on total amount of extracted glucose for diffusion, reverse iontophoresis (300 µA) and MHD (300 µA and 300 mT). The bars represent the average from at least 4 individual experiments and the error bars represent standard deviation of the mean. The values of individual experiments are indicated with the circles on top of the bar graphs. Dotted line on the bar graphs serves as a guide for the eye and represent the contribution of diffusion. The p-values were calculated using an unpaired t-test. (**b**) Amounts of actively extracted glucose after the effect of passive glucose diffusion is subtracted. This data indicates that MHD extraction is more effective that reverse iontophoresis also in shorter extraction times. Furthermore, most of the active extraction occurring at the early time points of the extraction.

To further investigate the glucose extraction from our porcine skin/hydrogel skin model, we varied the extraction time while keeping the extraction current at 300 µA (Fig. 3a,b). As expected, the amount of extracted glucose increased with increasing extraction time for both extraction methods (Fig. 3a). However, after the effect of passive glucose diffusion was subtracted (Fig. 3b), the actively extracted amount of glucose remained relatively constant at 0.31 ± 1.1 µg, 0.54 ± 0.86 µg, and 0.17 ± 0.36 µg when using iontophoresis for 1 min, 5 min, and 10 min, respectively. In contrast, MHD achieved a significant increase in the amount of actively extracted glucose from 0.52 ± 0.88 µg to 2.50 ± 0.37 µg when increasing the extraction time from 1 to 5 min. Interestingly, the amount of actively extracted glucose remained relatively constant when further increasing the extraction time from 5 to 10 min. Hence, most of the glucose extracted with active methods (reverse iontophoresis and MHD) occurred during the first 5 min. This may be related to time-dependent changes in concentration gradients causing back-diffusion of glucose towards the hydrogel during prolonged extraction (Fig. 3b). Similar effects have been observed by others with urea, potassium, and cysteine as analytes using reverse iontophoresis^44,50,51^, however, no comprehensive explanation of this effect currently exists.

### Skin damage assessment

**Figure 4:**
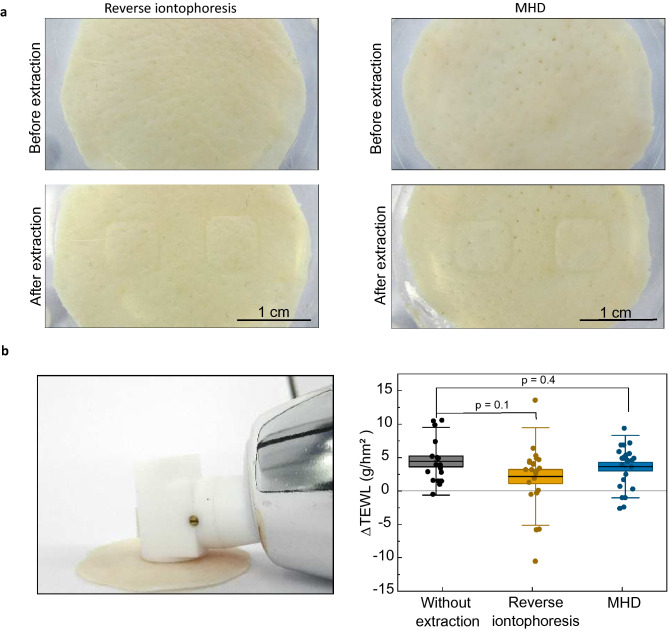
(**a**) Photographs of porcine skin samples used in this study either with reverse iontophoresis or MHD. No damage apart from minor imprints from the electrode wells were visible. (**b**) Skin water loss was measured at room temperature using a Tewameter before and after extraction (delta = after extraction-before extraction). The Tewameter readings measured after reverse iontophoresis (n = 22) and extraction with MHD (n = 23) were not statistically different from the values measured for samples where no active extraction was applied (n = 17). Individual data points are represented with circles and the boxes behind the data points represent the standard error. The thin, horizontal lines mark the mean values, and the error bars depict standard deviation of the dataset. The p-values were calculated using an unpaired t-test.

Potential damage to the skin induced by either MHD or reverse iontophoresis was investigated with trans-epidermal water loss measurements (TEWL)^52,53^ and visual inspection performed before and after the extraction (Fig. 4a). Average TEWL measurements after the extraction were slightly higher than before the extraction for MHD, reserve iontophoresis, and passive diffusion (Fig. 4b). Reverse iontophoresis showed higher average ΔTEWL when compared to MHD and passive diffusion. However, there was no statistical difference between the extraction and reference experiments, which indicates that the increase was caused by the highly hydrated conditions in the extraction cell. This implies that neither of the extraction methods affect the skin barrier function.

## Discussion

We presented a novel extraction method for interstitial fluid sampling that relies on MHD and the Lorentz force. Utilizing glucose as the analyte and a well-established substitute for human skin, the porcine ex vivo skin model, we compared the efficiency of MHD and reverse iontophoresis to passive diffusion under well-defined experimental conditions. The results showed that the efficiency of the MHD extraction method is superior to that of reverse iontophoresis. Using a 300 mT magnetic field we achieved a 13-fold increase in active glucose extraction. Furthermore, the amount of extracted glucose was proportional to the glucose concentration in the sample, which is important when considering potential applications in glucose sensing where the extracted amount of glucose in ISF is expected to correlate with the concentration of glucose in blood. Transepidermal water loss measurements before and after extraction showed no significant differences, implying that the extraction does not damage the skin permeability barrier. In this paper, we reported relatively high values of diffusion when compared to the active extraction. This indicates that the porcine skin grafts were probably more preamble than intact human skin. Thus, this study encourages more research into ex vivo models which could better represent the effect of microcirculation and barrier function of living skin.

The proposed technology can be optimized by exploring different electrode shapes, sizes, and distances, in addition to extraction current waveforms, to increase the glucose extraction rate obtained with MHD. Moreover, the magnet arrangements can be decreased in size if the magnets are moved closer to the skin surface. For example, when using a Neodymium magnet as small as 5 mm × 5 mm, a magnetic field of 265 mT is present at the surface of the magnet  (Fig. S7). Thus, the MHD technology has the potential to be applied in wearable devices for noninvasive glucose monitoring. These small magnets could be installed into a portable device such as a wrist band or sport watch with glucose sensitive electrodes.

Since the MHD technology is non-selective, these findings imply that the MHD method could potentially be used to extract other analytes present in ISF (e.g. Na^+^, K^+^, and lactate) which could be of interest in biomarker sensing. Furthermore, the extraction rate achieved with MHD could enable detection of sparse analytes, such as cortisol, that exist in interstitial fluid at concentrations below 1 µM^54^. Consequently, the presented MHD extraction method could be valuable to the development of wearable chemical sensors and biosensors utilizing interstitial fluid as the sample. Furthermore, by switching either the poles of the magnet or the direction of the current, the direction of the magnetohydrodynamic force can be reversed. Thus, this technology could potentially also be used to deliver molecules through the skin and into the human body.

In conclusion, we anticipate that the results presented in this work may encourage researchers to revisit the utilization of ISF as a sample for noninvasive on-body chemical sensing.

## Methods

### Synthesis of gelatin methacryloyl (GelMA)

GelMA was synthetized by functionalising gelatin from bovine skin (type B, Sigma Aldrich) with methacrylic anhydride (MEA, Sigma Aldrich) using a protocol adapted from ref.^55^. Briefly, 10% gelatin solution was prepared by dissolving gelatin powder into PBS (pH 7.4) at 50 °C. While vigorously stirring, 0.6 g of MEA per 1 g of gelatin was added dropwise into the gelatin solution and the reaction was stirred at 50 °C for 3 h. Unreacted MEA was removed by 5 min centrifugation at 2180 G followed by 5-day dialysis (10 kDa cut off) of the supernatant. Finally, GelMA was lyophilized for one week and stored at − 20 °C. The degree of functionalization (DOF) in GelMA synthesis was measured using an OPA (o-phthalaldehyde) assay^55^ and 86% DOF was achieved.

### Preparation of GelMA hydrogel

The protocol for GelMA hydrogel preparation was adapted from ref.^56^. GelMA hydrogel was prepared from 10% GelMA solution (in PBS, pH 7.4) with 0.15% of lithium phenyl-2,4,6-trimethylbenzoylphosphinate photoinitiatior (LAP). The mixture was placed in a hand casting mold (Mini-PROTEAN^®^ Tetra Handcast System, Biorad) with a 1.5 mm spacer and was polymerized with UV-C light (Carbon XM-120V, Green UV) for 5 min. The crosslinked GelMA was placed into PBS to remove residues of LAP. For doping the gel with glucose, the GelMA powder was dissolved into PBS with glucose and the hydrogels were made as previously described. Crosslinked GelMA hydrogels were then stored in PBS with glucose until used.

### Preparation of porcine skin

We purchased the porcine ears from a local eco farm (Kiven säästöpossu, Karkkila, Finland). We collected the ears from the slaughterhouse, where the ears remained at room temperature for 2 h after sacrificing the animals. Afterwards, the ears were transported on ice for 1 h. Then, the ears were washed with cold running water. From the posterior surface of each ear, 2 to 3 round samples of 4 cm diameter and between 600 and 700 µm thickness (Fig. S4) were dissected using a dermatome (Nouvag, Switzerland). Individual skin samples were wrapped in parafilm and stored at – 20 °C and were used within the next two weeks. Trans-epidermal water loss was measured before freezing and after defrosting to track damage of the skin during skin sample storage (Fig. S5). The measurements were done with a Tewameter^®^ (TM 300, Courage + Khazaka electronic GmbH, Germany) using the software (MPA WL version 1.3.1) according to the manufacturer’s instructions. An example of a Tewameter^®^ reading is shown in Fig. S5. After defrosting, the skin samples were equilibrated at room temperature (22 ± 1 °C) in PBS for 1.5 h with changing the buffer every 30 min. Extra buffer on the skin surface was removed by absorbing it with paper tissue before each experiment.

### Silver wire chlorination

Silver wires (diameter = 1.0 mm, Sigma Aldrich) were cleaned with 120-grit sandpaper and rinsed with water and 2-propanol. The clean wires were placed in a 1 M KCl bath and 300 µA current was passed through them for 3 h. After chlorination, the wires were rinsed with deionized water, dried gently with pressurized air, and stored protected from light.

### Experiments with extraction cell

All measurements and experiments were done at 22 ± 1 °C. A two-layer hydrogel skin model was constructed in an extraction chamber (Fig. S1) by carefully placing a piece of GelMA hydrogel 1.5 mm thick and 3 cm in diameter with a specific glucose concentration into the extraction chamber. A porcine skin sample (thickness: 600 to 700 µm; ø = 4 cm) was placed on top of the GelMA (care was taken to avoid trapping air between the layers). Then, the diffusion chamber was tightly sealed with plastic screws. Subsequently, the electrode wells were filled with 400 µl of PBS (pH 7.4, Sigma Aldrich) and the Ag/AgCl electrode wires were immersed into the buffer solution avoiding contact with the walls of the well. A current of 100, 200 or 300 µA was passed through the electrodes using a current source (Model 6220, Keithley Instruments). For MHD extraction, the set up was placed at the center and in between two or more magnets (neodymium magnets, Goliath, Supermagnete) which established a homogeneous magnetic field at the extraction site. The set of magnets were positioned carefully so that the magnetic field was perpendicular to the current field thereby causing the Lorenz force predominantly orthogonal to and pointing away from the skin surface. The two highest magnetic fields were obtained by placing the extraction chamber in the middle of an ensemble of four magnets (Fig. S1). The magnetic field strength was measured inside an empty electrode well with an AC/DC magnetic meter (PCE-MFM 3000). Trans-epidermal water loss was measured before and after extraction to track potential damage of the skin during extraction experiments. The water-loss measurements were conducted with a Tewameter^®^ (TM 300, Courage + Khazaka electronic GmbH, Germany).

### Determination of glucose concentration

The glucose concentration was measured using a protocol adapted from ref.^57^. Accordingly, 125 µl samples were taken from the electrode wells and mixed with 250 µl of reaction mixture containing 12.8 U/ml of glucose oxidase, 2.6 U/ml of horse radish peroxidase, and 0.13 mg/ml of o-dianisidine in PBS (pH 7.4). This mixture was incubated at 37 °C for exactly 30 min. Immediately thereafter, 250 µl of 12% sulfuric acid was added to the mixture. The light absorbance of the reaction product, oxidized o-dianisidine, was measured at 540 nm using a UV–VIS spectrophotometer (UV-1900, Shimadzu) and was compared to the standard curve (Fig. S6).

## Supplementary Information

Supplementary Information.
