# Supplementary material for: Sampling of fluid through skin with magnetohydrodynamics for noninvasive glucose monitoring
Source: Sci Rep. 2021 Apr 7;11:7609. doi: 10.1038/s41598-021-86931-7 (PMC8027418; doi:10.1038/s41598-021-86931-7)
Supplement: Supplementary file 1 — Supplementary Information. [file 41598_2021_86931_MOESM1_ESM.docx]

Supplementary Information

**Sampling of fluid through skin with magneto-hydrodynamics for noninvasive glucose monitoring**

Tuuli A. Hakala^1,#^, Alejandro García Pérez^1,5,#^, Melissa Wardale^1^, Ida A. Ruuth^1^, Risto T. Vänskä^1^, Teemu A. Nurminen^1^, Emily Kemp^1^, Zhanna A. Boeva^1,2^, Juha-Matti Alakoskela^1,3^, Kim Pettersson-Fernholm^1,4^, Edward Hæggström ^1,5^, and Johan Bobacka ^1,2,^ *

^#^ These authors contributed equally to the work

^1^ *Glucomodicum Ltd, A.I. Virtasen Aukio 1, 00560 Helsinki, Finland*

*^2^Laboratory of Molecular Science and Engineering, Faculty of Science and Engineering, Åbo Akademi University, Biskopsgatan 8, 20500 Turku/Åbo, Finland*

*^3^Skin and Allergy Hospital, Meilahdentie 2, 00250 Helsinki, Finland*

^4^ *Nefrologian poliklinikka, Helsinki University Hospital, Haartmaninkatu 4, 00029 Helsinki, Finland*

^5^ *Department of physics, University of Helsinki,* *Gustaf Hällströmin katu 2, 00560 Helsinki, Finland*

^*^ Corresponding author: [johan.bobacka@abo.fi](mailto:johan.bobacka@abo.fi)

**Chemicals**

D(+)-glucose (#1.08337) was purchased from Merck. Gelatin from bovine skin (#G9382), glucose oxidase (#G7141), peroxidase from horseradish (#P8250-5KU), PBS pH 7.4 (#P3813), methacrylic anhydride (#276685), and o-dianisidine dihydrochloride (#D3252) were purchased from Sigma Aldrich. Sulfuric acid (#20700.290) was purchased from VWR.

**Figures**


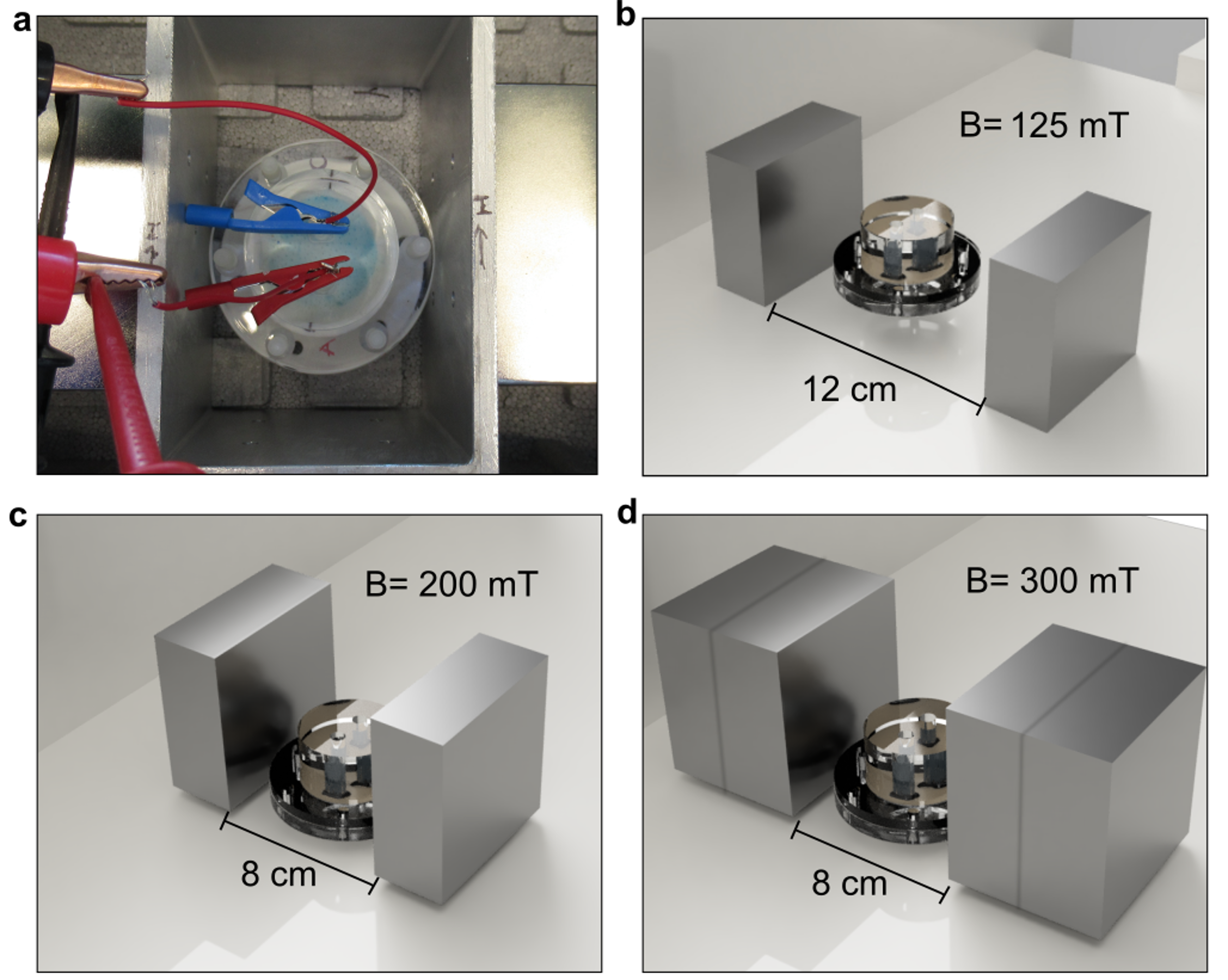


**Supplementary Figure S1. MHD extraction set up.** **a**) Picture of MHD set up with Ag/AgCl wires connected.  **b**)-**d**) 3D illustrations of the magnet configurations for 125 mT using 2 magnets 12cm apart, **b**) 200 mT using 2 magnets 8 cm apart, **c**) and 300 mT using 4 magnets 8 cm apart **d**).


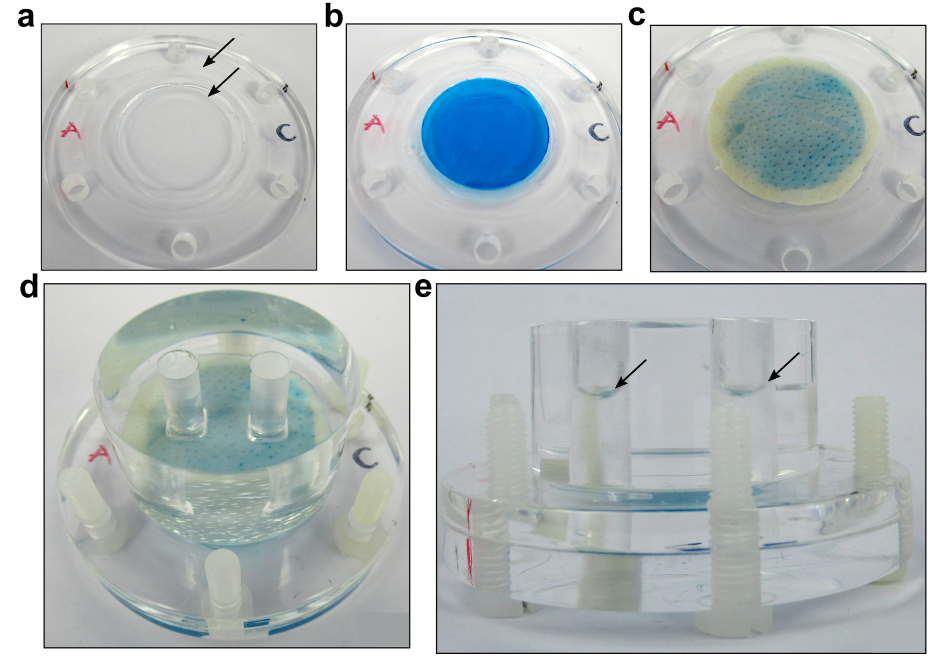


**Supplementary Figure 2.**  **Constructing the extraction set up**. **a**) bottom of the well has a 3 cm (diameter) indentation for the GelMA hydrogel and another 4 cm (diameter) indentation for skin (indicated with arrows). **b**) 1.5 mm thick glucose saturated hydrogel (here colored blue to increase the photographic contrast) is placed at the bottom, and **c**) dermatomed porcine skin slab (600 to 700 µm thickness) on top. **d**) This two-layer skin model is sealed into the extraction cell with plastic screws. **e**) Finally, 400 µl of buffer is placed into the electrode wells and the level of buffer (indicated with arrows) is checked to ensure good seal of the cell.


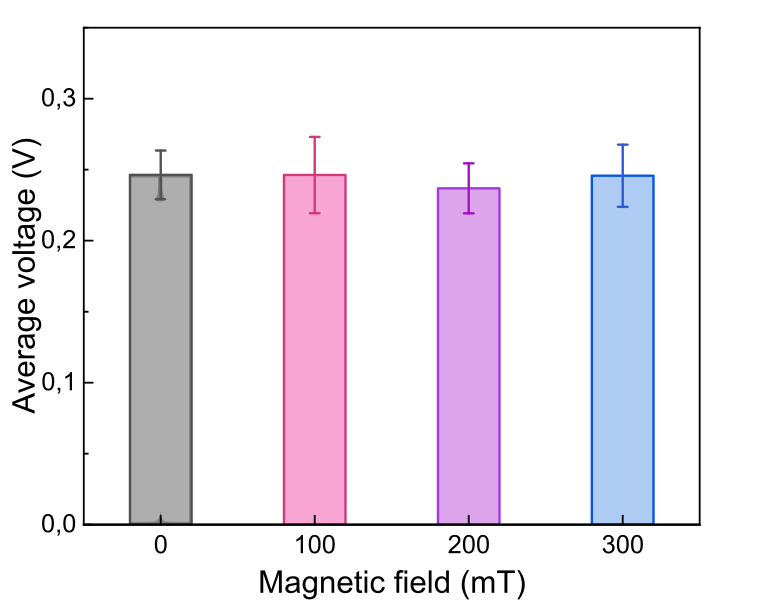


**Supplementary Figure 3. Voltage during MHD extraction.** Average voltage during extraction with a current of 300 µA and different magnetic field strengths.


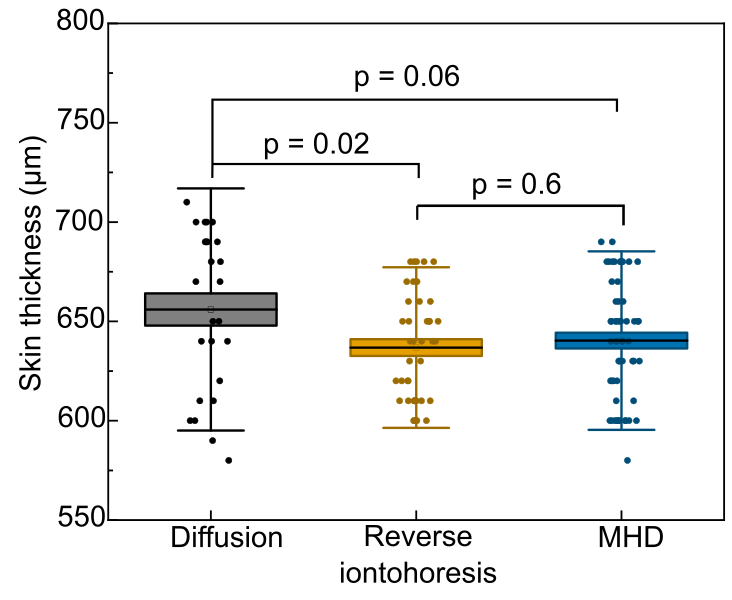


**Supplementary Figure 4. Thickness of skin samples used for extraction studies.** Skin samples were dermatomed to nominally 600 µm thickness and the thickness of each sample was measured after dermatoming using digital pocket caliber (230.207, Scala). Skin samples were assigned randomly to each experiment. The graph shows the thickness of all the samples used for diffusion, reverse iontophoresis and MHD experiments (dots). The horizontal line in the middle of the box represents the average, the box represents the standard error, and the whiskers represent the standard deviation. The average of all the skin samples was 640 µm.


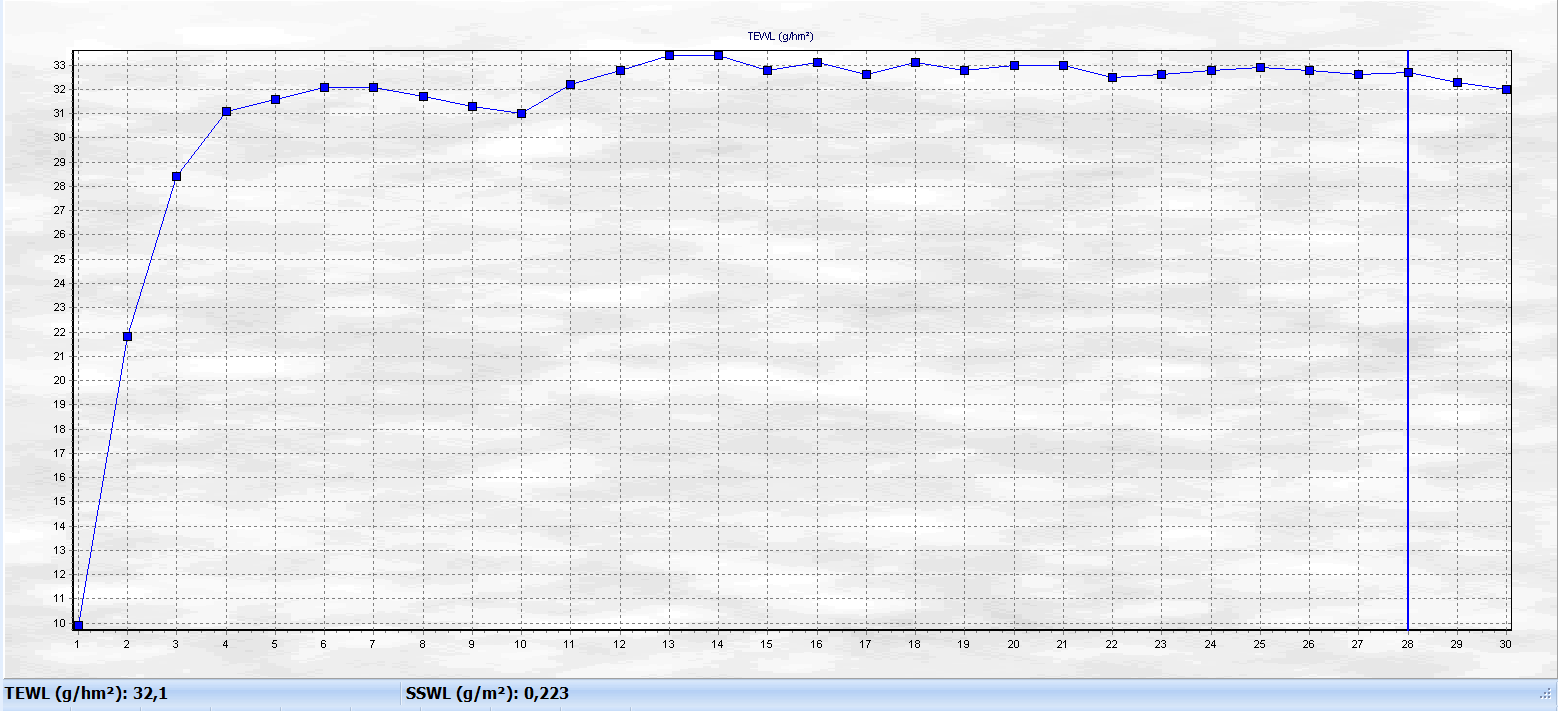


**Supplementary Figure 5. Example of Tewameter raw data.** The graph shows on the x-axis the measurement count and on the y-axis the TEWL (g/hm^2^) readings. The recorded TEWL value (indicated with blue vertical line) was automatically determined by the manufacturer’s software (MPA WL FB) so that it minimizes the standard deviation.


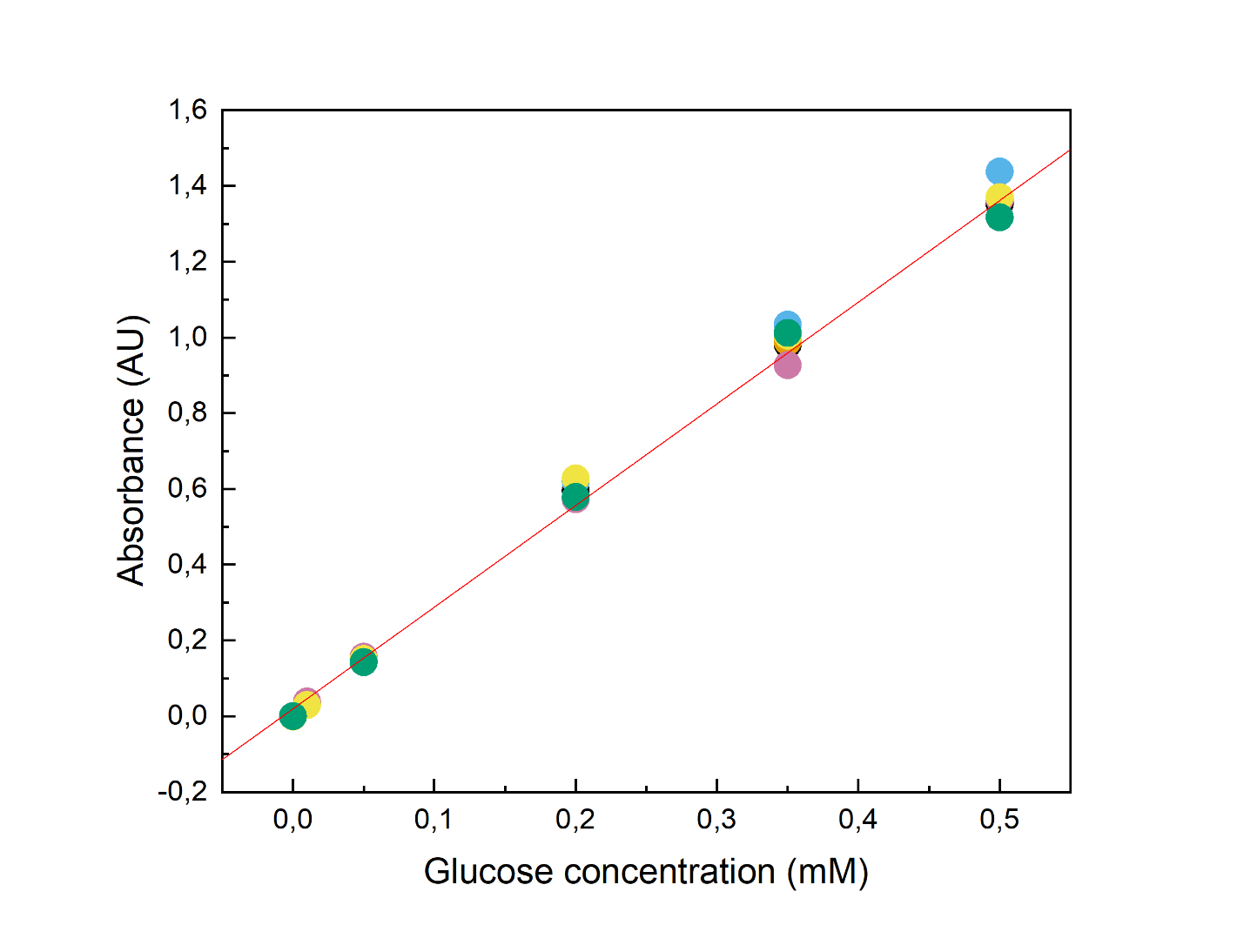


**Supplementary Figure 6.** Standard curve was reproduced for each glucose extraction analysis and the measured values lay within the linear region of the calibration curve (glucose concentrations between 0.01 mM to 0.6 mM). Different colors indicate individual experiments.


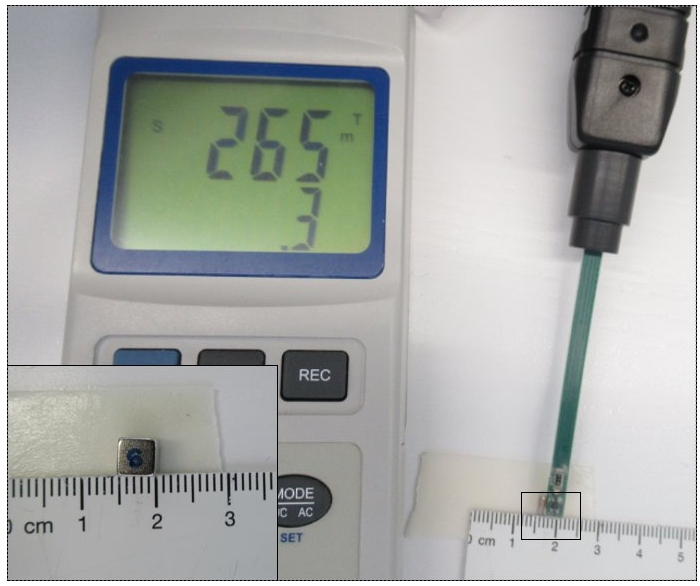


**Supplementary Figure 7.** Magnetic field measured at the south pole of 5 mm × 5 mm neodymium magnet using AC/DC magnetic meter (PCE-MFM 3000).
